# ECM stiffness regulates lung fibroblast survival through RasGRF1-dependent signaling

**DOI:** 10.1016/j.jbc.2025.108161

**Published:** 2025-01-08

**Authors:** Elizabeth Monaghan-Benson, Julien Aureille, Christophe Guilluy

**Affiliations:** 1 Department of Molecular Biomedical Sciences, College of Veterinary Medicine, North Carolina State University, Raleigh, North Carolina, USA; 2 Institute for Advanced Biosciences Centre de recherche UGA, INSERM U1209, CNRS UMR, Grenoble, France

**Keywords:** mechanotransduction, Ras, extracellular matrix, signaling, apoptosis, ERK, AKT, FOXO, Bcl-2 family

## Abstract

Extracellular matrix stiffness is one of the multiple mechanical signals that alter cellular behavior. During studies exploring the effect of matrix rigidity on lung fibroblast survival, we discovered that enhanced survival on stiff substrates is dependent on elevated Ras activity, owing to the activation of the guanine nucleotide exchange factor, RasGRF1. Mechanistically, we found that the increased Ras activity lead to the activation of both the AKT and ERK pathways. Pharmacological inhibition of AKT or ERK signaling attenuates the elevated survival observed on stiff substrates. AKT signaling regulates the phosphorylation and inactivation of the transcription factor FOXO3a. RNAi experiments demonstrate that FOXO3a activity is critical for the cell death observed on soft substrates. Additionally, downregulation of FOXO3a activity on stiff substrate leads to the degradation of the proapoptotic protein Bim. Depletion of Bim increased the survival of cells on soft substrates. Together, our data show that enhanced matrix stiffness activates a RasGRF1/Ras signaling cascade that regulates the activity of AKT and ERK-dependent FOXO3a and Bim expression to alter cell survival.

Tissue growth and homeostasis is governed by a number of biochemical and biophysical factors within the cellular microenvironment (1, 2, 3). Among the biophysical factors is matrix stiffness, which impacts a diverse range of cellular processes including proliferation, migration, differentiation, and survival (4).The optimum matrix rigidity for survival is largely cell type–specific as the elastic modulus of living tissues spans multiple orders of magnitude (5). Our understanding of how cells transduce the mechanical inputs from their microenvironments into the biochemical signaling networks that regulate cellular behavior remain incompletely understood.

Ras GTPases act as molecular switches, active when GTP-bound and inactive when GDP-bound. Guanine nucleotide exchange factors (GEFs) activate Ras proteins by catalyzing the exchange of GDP for GTP (6). Upon activation, Ras binds and activates effector proteins modulating signaling networks that regulate many aspects of cell behavior, including growth, proliferation, differentiation, and survival (7). The number Ras effector pathways is considerable, but the MAPK/ERK and PI3K/AKT cascades are two of the best characterized downstream effectors of Ras signaling (8).

Ras signaling promotes cell survival by activating prosurvival BCL2 proteins (BCL2, BCL-x_L_, and MCL1) and repressing prodeath proteins (BAD, BIM, BMF, and PUMA) (9). Bim is a proapoptotic BH3-only member of the Bcl-2 family. Activity of Bim is largely regulated by its protein expression level. Bim expression is induced by activation of the FOXO family of transcription factors, a downstream target of the AKT pathway (10, 11). Conversely, Bim protein expression is downregulated by proteasomal degradation following ERK-dependent phosphorylation and ubiquitination (12).

Normal fibroblasts cultured on a flexible matrix show enhanced levels of apoptosis (13). Interestingly, H-Ras transformed fibroblasts did not show enhanced rates of apoptosis on flexible substrates. Recent studies have demonstrated that K-ras allows cells to sense the compliance of the extracellular matrix (ECM) and activate transcriptional events to promote proliferation and self-renewal (14). Although these results suggest a role for Ras in regulating mechanical survival, the precise molecular mechanisms that regulate this process remain poorly defined.

In the present study, we sought to determine the mechanism by which ECM stiffness regulates cell survival. Our results show that enhanced ECM stiffness results in an increase in Ras activity owing to the activation of the Ras GEF RasGRF1. This increase in Ras activity activates the PI3K/AKT and MAPK/ERK pathways resulting in phosphorylation of the transcription factor FOXO3a and the degradation of the proapoptotic factor Bim.

## Results

**Figure 1:**
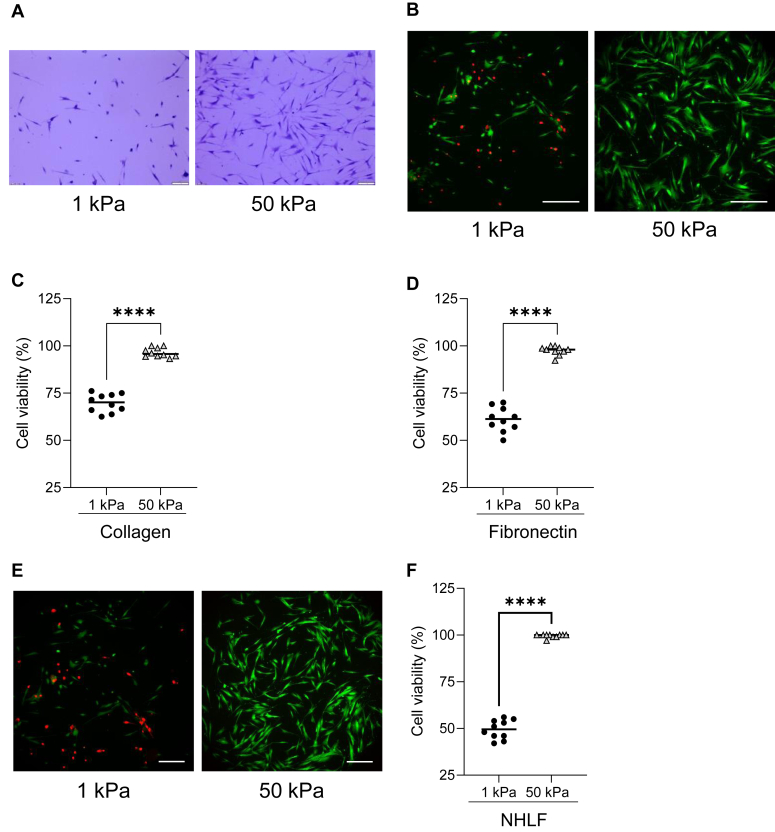
**Enhanced substrate rigidity induces lung fibroblast survival.***A*, MRC5 cells were cultured on soft (1 kPa) and stiff (50 kPa) substrates coated with collagen (20 μg/ml) for 24 h. Cells were then fixed and stained with crystal violet before visualization by light microscopy. MRC5 cells were cultured on soft (1 kPa) and stiff (50 kPa) substrates coated with (*B* and *C*) collagen (20 μg/ml) or (*D*) FN (20 μg/ml) for 24 h. Cell viability was assessed with a live/dead assay. *E* and *F*, NHLF were cultured on soft (1 kPa) and stiff (50 kPa) substrates coated with collagen (20 μg/ml) for 24 h before cell viability was assessed with a live/dead assay. A minimum of 300 cells per condition were counted. The scale bar represents 50 μm ∗∗∗ indicates *p* < 0.001 ∗∗∗∗ indicates *p* < 0.0001 as determined by Student’s *t* test. NHLF, normal human lung fibroblast.

The Young’s modulus of healthy lung tissue ranges between 1 kPa and 5 kPa (15), while fibrotic lung tissue measures stiffness values of 20 kPa to 100 kPa (16). For this study, we used a lung fibroblast cell line, MRC5, and used 1 kPa hydrogels to represent a soft matrix (within the physiological range for the healthy lung) and 50 kPa hydrogels to represent a stiff matrix (with the pathological range for the fibrotic lung). To determine if substrate stiffness impacted lung fibroblast number, cells were plated onto soft and stiff substrates and then stained 24 h later with crystal violet for visualization. The crystal violet staining revealed fewer cells present on the soft substrates than the stiff substrates (Fig. 1*A*).

To test the impact of matrix stiffness on cell survival, MRC5 cells were plated onto collagen-coated “soft” (1 kPa) or “stiff” (50 kPa) gels and incubated for 24 h. The cells were then assessed for viability using a live/dead assay kit and visualized by fluorescence microscopy. Cells plated on the soft (1 kPa) substrates showed a ∼40% increase in cell death when compared to the cells plated on the stiff (50 kPa) substrate (Fig. 1, *B* and *C*). To evaluate whether ECM composition affected survival in response to matrix rigidity, viability was analyzed in cells plated on fibronectin. Similar results were observed when cells were plated on soft and stiff gels coated with fibronectin. Cells plated on the soft gels showed ∼45% increase in cell death when compared to cells plated on the stiff gels (Fig. 1*D*). Since cellular signaling and behavior can be affected by ECM coating concentration, we examined the relative collagen and fibronectin coating levels on the soft and stiff hydrogels (Fig. S1). For the collagen experiment we used an Alexa Fluor 488–conjugated collagen antibody and analyzed its ability to bind soft and stiff hydrogels coated with 20 μg/ml collagen. We identified random regions of interest and calculated a mean fluorescence intensity on both the soft and stiff collagen coated hydrogels. The results demonstrate similar fluorescence intensities on the soft and stiff substrates, indicating similar levels of collagen coating. For the analysis with FN coating, we used an Alexa Fluor 488–conjugated FN and analyzed a series of fibronectin coating densities microscopically. We identified random regions of interest and calculated a mean fluorescence intensity for each of the coating concentrations on both the soft and stiff hydrogels. The results demonstrate similar fluorescence intensities on the soft and stiff substrates, indicating similar levels of FN coating. Therefore, any differences observed on the soft and stiff substrates are not due to a difference in ECM-coating concentrations. Collectively, these results indicate it is the rigidity of the matrix and not the ECM molecule promoting the change in cell survival.

To extend our analysis, we tested the survival response of primary normal human lung fibroblasts (NHLFs) on soft (1 kPA) and still (50 kPA) substrates (Fig. 1, *E* and *F*). Similar to the results with the MRC5 cells, we found that NHLF plated on soft substrates showed ∼50% increase in cells death when compared to plating on stiff gels. These data recapitulate the finding that increased substrate stiffness enhances lung fibroblast survival.

**Figure 2:**
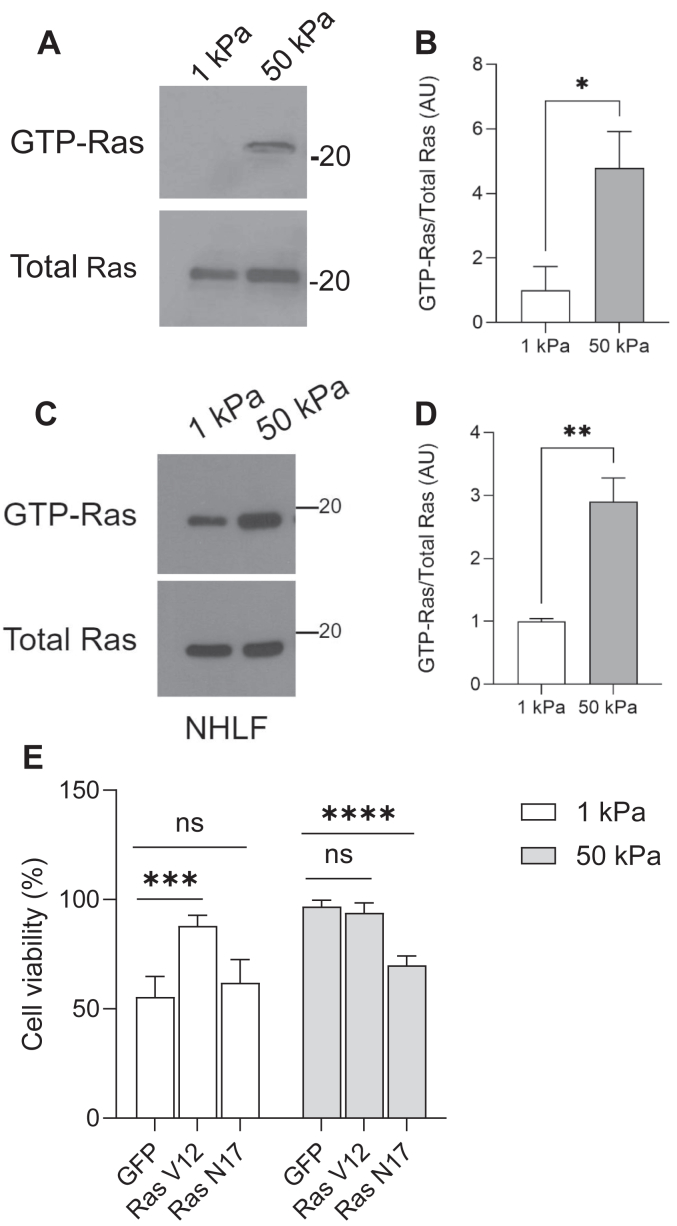
**Enhanced substrate rigidity induces Ras-dependent cell survival.***A*, MRC5 cells were cultured on 1 kPA and 50 kPa collagen-coated (20 μg/ml) substrates for 24 h. Cells were lysed and then analyzed for the activation of Ras measured by a GST-RBD pull-down assay as described under Experimental procedures. *B*, quantification of GTP-Ras/total Ras levels compiled from three replicates. *C*, NHLF were cultured on 1kPA and 50 kPa collagen-coated (20 μg/ml) substrates for 24 h. Cells were lysed and then analyzed for the activation of Ras measured by a GST-RBD pull-down assay as described under Experimental procedures. *D*, quantification of GTP-Ras/total Ras levels compiled from three replicates. *E*, MRC5 cells were adenovirally infected with GFP, RasV12, or RasN17 for 24 h before replating onto collagen-coated (20 μg/ml) soft and stiff substrates for an additional 24 h. Cell viability was then analyzed using a live/dead assay. Three hundred cells were counted for each treatment condition. ∗∗∗ indicates *p* < 0.001 ∗∗∗∗ indicates *p* < 0.0001 as determined by Student’s *t* test. GST, glutathione-*S*-transferase; NHLF, normal human lung fibroblast; RBD, Raf1-binding domain.

Ras GTPases are well-known regulators of cell survival. To determine if Ras activity is affected by substrate stiffness, GTP-Ras levels were analyzed on MRC5 cells plated on soft and stiff substrates revealing that Ras-GTP levels were higher on cells plated on stiff substrates than cells plated on soft substrates (Fig. 2, *A* and *B*). To evaluate whether Ras activity was also impacted in primary lung fibroblasts, we analyzed GTP-Ras levels in NHLF as well (Fig. 2, *C* and *D*). Again, we found higher levels of GTP-Ras in the cells plated on stiff substrates than cells plated of soft substrates. To determine whether the enhanced survival of the cells plated on the stiff substrates is dependent on Ras activity, we used adenovirally delivered Ras mutants, dominant/negative Ras N17 and constitutively active Ras V12 to regulate Ras activity. We found that expression of the dominant negative Ras N17 abbrogated the enhanced survival observed on stiff substrates (Fig. 2*E*), while expression of constitutively active Ras V12 increased survival of cells plated on soft substrates (Fig. 2*E*). These data are consistent with the idea that Ras activity is critical in driving the enhanced survival observed on stiff substrates.

**Figure 3:**
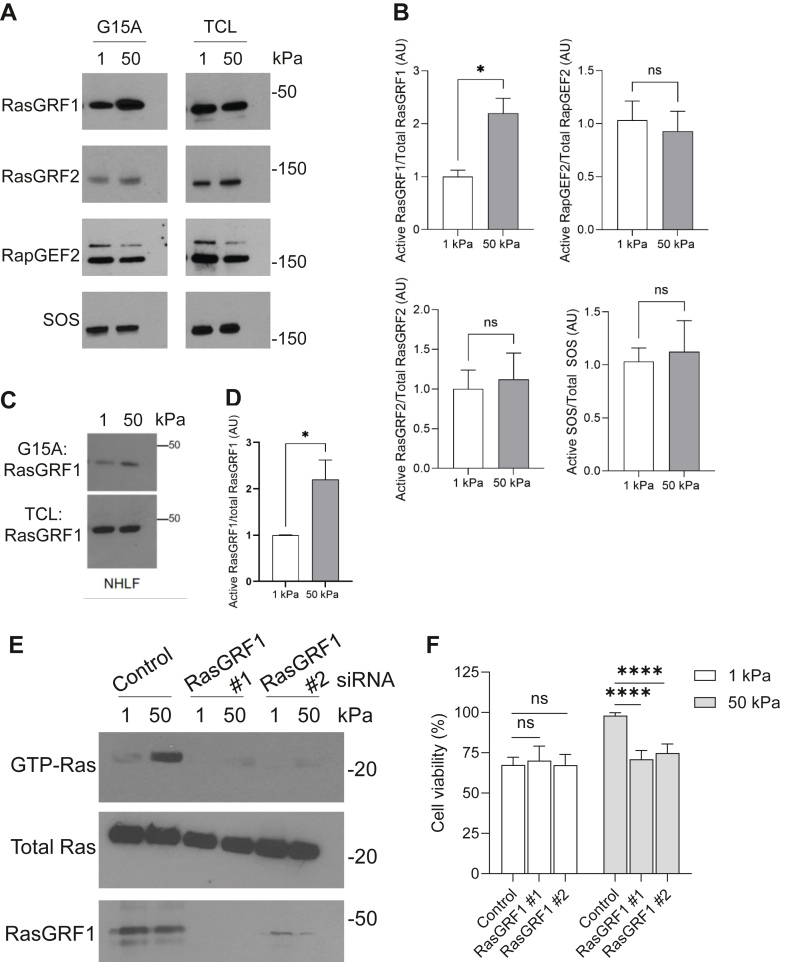
**RasGRF1 mediates enhanced Ras activity in response to substrate stiffness.***A*, MRC5 cells were cultured on 1 kPa and 50 kPa collagen-coated substrates for 24 h. Cells were then lysed and the activity of Ras GEFs was determined using the GST-Ras^G15A^ pull-down assay followed by immunoblotting. *B*, quantification of active/total protein levels complied from three replicates. *C*, NHLF were cultured on 1 kPa and 50 kPa collagen-coated substrates for 24 h. Cells were then lysed and activity of RasGRF1 was determined using the GST-Ras^G15A^ pull-down assay followed by immunoblotting. *D*, quantification of active Ras GRF1/total RasGRF1 protein levels complied from three replicates. *E* and *F*, MRC5 cells were transfected with control, RasGRF1 #1, or RasGRF1 #2 siRNA for 72 h. After 48 h, the cells were then replated onto 1 kPa and 50 kPa collagen-coated substrates for an additional 24 h. *B*, cell lysates were analyzed for levels of active Ras through GST-Ras-RBD pull-down assay and by Western blotting for levels of RasGRF1. *C*, cell viability was analyzed using live/dead assays. Cells were visualized by microscopy and 300 cells were counted per condition. ∗∗∗ indicates *p* < 0.001 ∗∗∗∗ indicates *p* < 0.0001 as determined by Student’s *t* test. GEF, guanine nucleotide exchange factor; GST, glutathione-*S*-transferase; RBD, Raf1-binding domain.

GEFs increase the activity of Ras by promoting the exchange of GDP for GTP (17). We wanted to determine if Ras GEF activity was affected by substrate stiffness. To look for activation of GEFs, we performed affinity pulldown assays with a nucleotide-free Ras mutant, Ras G15A. This analysis revealed that stiff substrates activate RasGRF1, but had no effect on the activities of several other GEFs including RasGRF2 (Fig. 3, *A* and *B*). To evaluate whether activation of RasGRF1 was observed in primary lung fibroblast in response to enhanced matrix stiffness, we again performed a nucleotide-free Ras G15A pulldown in NHLF cells (Fig. 3, *C* and *D*) revealing an increase in active Ras GRF1 on the rigid substrate, recapitulating the results found in the MRC5 cells. To determine if RasGRF1 is responsible for Ras activation on stiff substrates, we used siRNA to deplete RasGRF1 expression (Fig. 3*E*). Treatment with RasGRF1 siRNA inhibited the activation of Ras observed on stiff substrates (Fig. 3*E*). Furthermore, knockdown of RasGRF1 attenuated the enhanced survival of cells plated onto stiff substrates, while having no effect on the survival of cells plated on soft substrates (Fig. 3*F*). These data suggest that RasGRF1 regulates stiffness based cell survival through the regulation of Ras activity.

**Figure 4:**
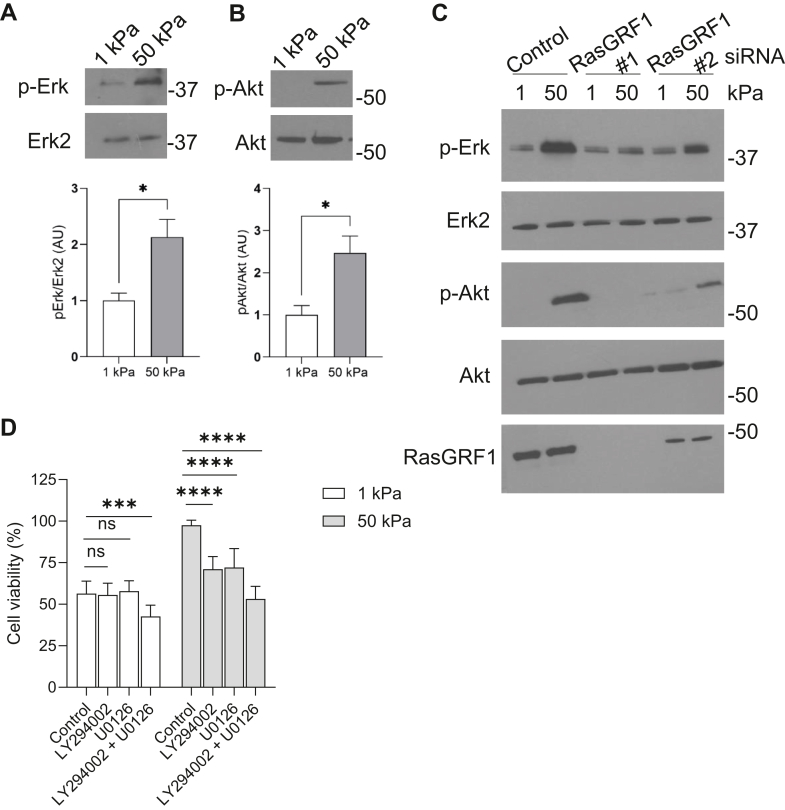
**RasGRF1 regulates phosphorylation of Erk and Akt in response to substrate stiffness.** MRC5 cells were cultured on 1 kPa and 50 kPa collagen-coated substrates for 24 h. Cells were lysed and analyzed for levels of (*A*) p-Erk and (*B*) p-Akt by Western blotting. Quantification of active/total protein levels complied from 3 replicates. *C*, MRC5 cells were transfected with control, RasGRF1 #1, or RasGRF1 #2 siRNA for 48 h. Cells were then replated onto collagen-coated 1 kPa and 50 kPa substrates for an additional 24 h. Cells were lysed and levels of p-Erk, Erk2, p-Akt, Akt, and RasGRF1 were analyzed by Western blot. *D*, MRC5 cells were plated onto collagen-coated 1 kPA and 50 kPA substrates in the presence of U0126 (5 μM) or LY294002 (10 μM) or both U0126 and LY294002 for 24 h. Cell viability was determined through a live/dead assay. Cells were visualized by microscopy, and 300 cells per condition were counted. ∗∗∗ indicates *p* < 0.001 ∗∗∗∗ indicates *p* < 0.0001 as determined by Student’s *t* test.

Ras activation regulates a number of downstream signaling pathways including the MEK/ERK and PI3K/AKT survival pathways. Cells plated on stiff substrates were observed to display higher levels of p-ERK and p-AKT than cells plated on soft substrates (Fig. 4, *A* and *B*). To test the idea that p-ERK and p-AKT are activated downstream of RasGRF1 mediated Ras activity we used siRNA to deplete RasGRF1 expression (Fig. 4*C*). siRNA knockdown of RasGRF1 attenuated the activity of p-ERK and p-AKT observed on stiff substrates (Fig. 4*C*). To determine if activation of ERK and AKT are required for the enhanced cell survival seen on stiff substrates, inhibitors of the MEK–ERK pathway (U0126) and the PI3K/AKT pathway (LY294002) were used. Cells treated with U1026 or LY294002 showed a reduction in survival when plated on stiff substrates (Fig. 4*D*) with an additive effect observed when cells were treated with both U0126 and LY294002 (Fig. 4*D*). These results are consistent with the idea that MEK/ERK and PI3K/AKT signaling are required for the enhanced survival on rigid substrates.

**Figure 5:**
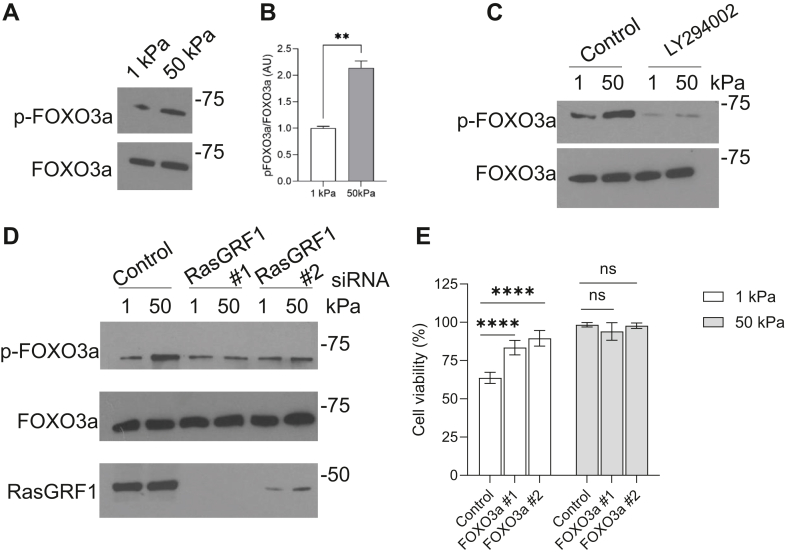
**FOXO3a is phosphorylated in response to increased substrate stiffness.***A*, MRC5 cells were cultured on 1 kPa and 50 kPa hydrogels coated with 20 μg/ml collagen for 24 h. Cells were lysed and lysates examined for levels of p-FOXO3a and FOXO3a. *B*, quantification of pFOXO3a/FOXO3alevels complied from three replicates. *C*, MRC5 cells were cultured for 24 h on 1 kPA and 50 kPa collagen-coated substrates in the presence of LY294002 (10 μM). Cells were lysed and analyzed for levels of p-FOXO3a and FOXO3a by Western blot. *D*, MRC5 cells were transfected with control, RasGRF1 #1, or RasGRF1 #2 siRNA for 72 h. After 48 h cells were then replated onto 1 kPa and 50 kPa collagen-coated substrates for an additional 24 h. Cells were lysed and evaluated for levels of p-FOXO3a, FOXO3a, and RasGRF1 by Western blot. *E*, MRC5 cells were transfected with control, FOXO3a #1, or FOXO3a #2 siRNA for 48 h. Cells were then replated onto collagen-coated substrates for an additional 24 h before a live/dead assay was performed. Cells were visualized by microscopy and 300 cells/condition were counted. ∗∗∗ indicates *p* < 0.001 ∗∗∗∗ indicates *p* < 0.0001 as determined by Student’s *t* test.

One downstream target of the AKT signaling pathway is the transcription factor Forkhead box protein O3 (FOXO3a), a major regulator of proapoptotic signaling (18). Regulation of FOXO proteins is achieved by changes in localization, with transcriptional activity occurring with nuclear localization (19). Phosphorylation of FOXO3a by AKT leads to its nuclear exclusion and degradation, resulting in the suppression of FOXO3a′s transcriptional activity (20). MRC5 cells were plated onto soft and stiff collagen coated gels and levels of p-FOXO3a were analyzed. Enhanced levels of p-FOXO3a were observed in cells plated on stiff substrates, suggesting that FOXO3a is not transcriptionally active (Fig. 5, *A* and *B*). Treatment with the PI3K inhibitor, LY294002, abrogated the phosphorylation of FOXO3a on stiff substrates (Fig. 5*C*). To determine if Ras activity was regulating FOXO3a phosphorylation, RasGRF1 levels were reduced through siRNA treatment and p-FOXO3a levels were examined by Western blotting. P-FOXO3a levels were greatly reduced in the absence of RasGRF1, suggesting that Ras activity is required for the phosphorylation of FOXO3a in response to matrix stiffness (Fig. 5*D*). To determine in FOXO3a is required for changes in cell survival in response to matrix stiffness, siRNA was used to deplete FOXO3a levels and viability was assessed through a live/dead assay. Knockdown of FOXO3a reduces the amount of cell death on soft substrates (Fig. 5*E*). These results are consistent with the idea that FOXO3a transcriptional activation modulates survival in response to substrate stiffness.

**Figure 6:**
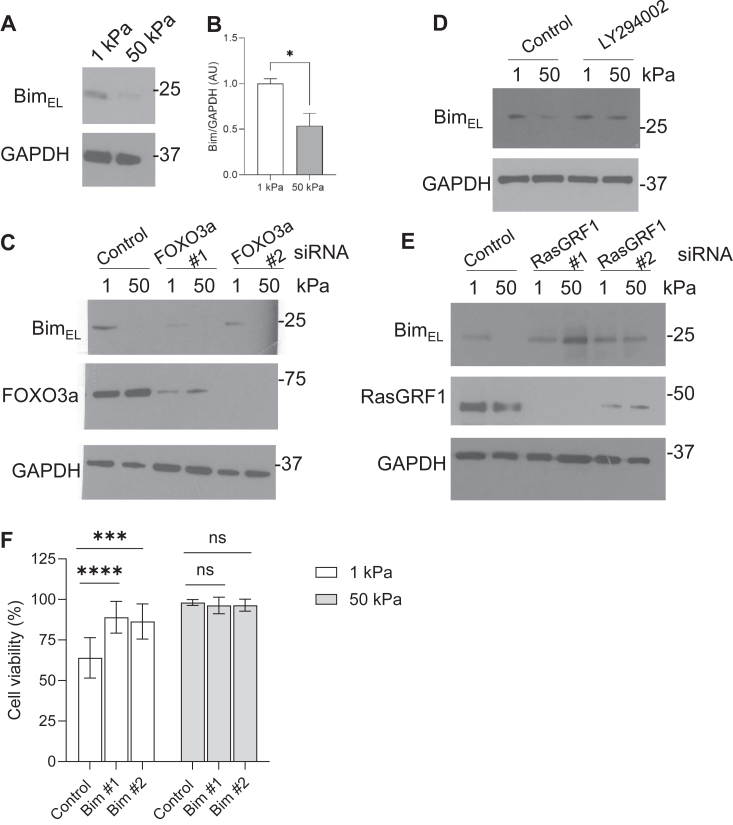
**Expression of BimEL decreases with increased substrate rigidity.***A*, MRC5 cells were cultured on 1 kPa and 50 kPa hydrogels coated with 20 μg/ml collagen for 24 h. Cells were lysed and lysates analyzed for expression of BimEL by Western blotting. *B*, quantification of BimEL/GAPDH levels complied from three replicates. *C*, MRC5 cells were transfected with control, Foxo3a #1, or Foxo3a #2 siRNA for 72 h. After 48 h, cells were replated onto 1 kPa and 50 kPa collagen-coated substrates for an additional 24 h. Cells were lysed and evaluated for levels of BimEL, FOXO3a, and GAPDH by Western blot. *D*, MRC5 cells were plated onto collagen-coated 1 kPA and 50 kPA substrates in the presence of LY294002 (10 μM) for 24 h. Cells were lysed and lysates probed with antibodies against BimEL and GAPDH. *E*, MRC5 cells were transfected with control, RasGRF1 #1, or RasGRF1 #2 siRNA for 72 h. After 48 h, cells were then replated onto 1 kPa and 50 kPa collagen-coated substrates for an additional 24 h. Cells were lysed and evaluated for levels of BimEL, RasGRF1, and GAPDH by Western blot. *F*, MRC5 cells were transfected with control, Bim #1, or Bim #2 siRNA for 72 h. Cells were then replated onto collagen-coated substrates for an additional 24 h before a live/dead assay was performed. Cells were visualized by microscopy and 300 cells/condition were counted. ∗∗∗ indicates *p* < 0.001 ∗∗∗∗ indicates *p* < 0.0001 as determined by Student’s *t* test.

FOXO3a is a known transcriptional activator of the proapoptotic protein Bim (11). To asses if Bim expression is regulated by substrate rigidity, we analyzed Bim protein levels in cells plated on soft and stiff substrates by Western blotting. Cells plated on 1 kPa soft substrates showed increased BimEL levels compared to cells plated on 50 kPa stiff substrates (Fig. 6, *A* and *B*). To determine if Bim expression is regulated by FOXO3a in response to matrix rigidity, we analyzed Bim levels in FOXO3a knockdown cells plated on soft and stiff substrates. FOXO3a knockdown reduced the levels of BimEL expressed in cells on soft substrates (Fig. 6*C*). Additionally, inhibition of AKT signaling with LY294002 raised BimEL levels in cells plated on rigid substrates (Fig. 6*D*). Furthermore, inhibition of Ras activity by knockdown of RasGRF1 was sufficient to raise BimEL levels on stiff substrates (Fig. 6*E*). To examine the role of Bim in cell survival on soft and stiff substrates a cell viability assay was performed on cells treated with Bim siRNA. Knockdown of Bim restored cell viability in cells plated on soft substrates (Fig. 6*F*). These results suggest that inhibition of Bim signaling is required for the enhanced survival of cells on stiff matrix.

**Figure 7:**
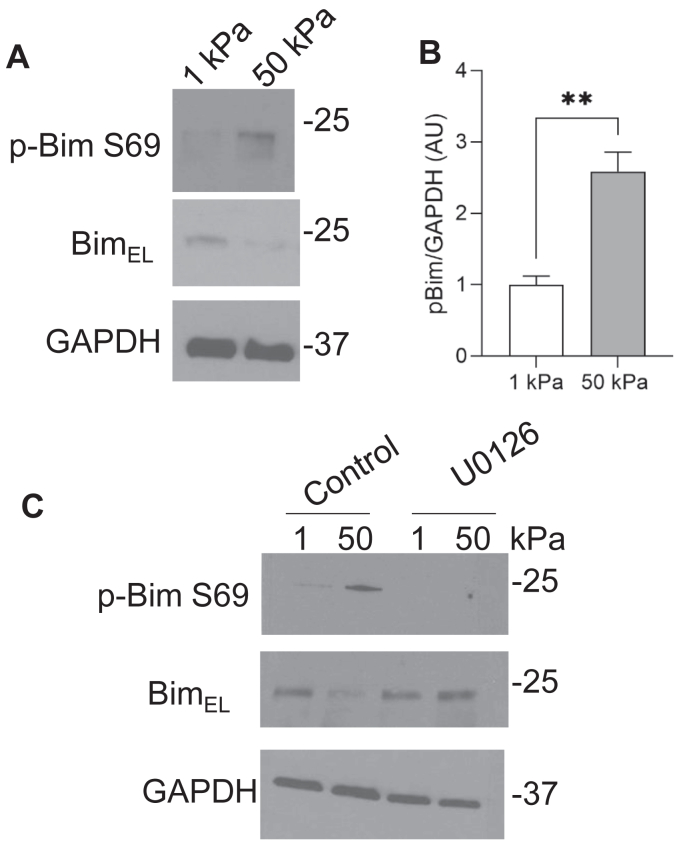
**Bim is phosphorylated at S69 in response to substrate rigidity.***A*, MRC5 cells were cultured on 1 kPa and 50 kPa hydrogels coated with 20 μg/ml collagen for 24 h. Cells were lysed and lysates analyzed for expression of p-Bim S69, BimEL, and GAPDH by Western blotting. *B*, quantification of p-Bim/GAPDH protein levels complied from three replicates. *C*, MRC5 cells were plated onto collagen-coated 1 kPA and 50 kPA substrates in the presence of U0126 (5 uM) for 24 h. Cells were lysed and lysates probed with antibodies against p-Bim S69, BimEL, and GAPDH.

In Figure 4, we found that the MEK-ERK inhibitor U0126 inhibited enhanced survival of cells on stiff substrates. Interestingly, Bim has been shown to be degraded by the proteasome upon ERK phosphorylation at S69 (12, 21, 22). We analyzed lysates from cells plated on soft and stiff matrix and found that while BimEL levels were low in cells plated on stiff substrates p-Bim S69 levels were increased in these same cells when compared to cells plated on soft substrates (Fig. 7, *A* and *B*). To determine if this phosphorylation of BimEL at S69 was downstream of MEK-ERK, cells were treated with U0126. Upon treatment of stiff cells with U0126 the levels of p-Bim S69 decreased while levels of total BimEL increased (Fig. 7*C*). Collectively, these data suggest that Bim is phosphorylated by the MEK/ERK pathway when cells are cultured on stiff substrates, leading to its degradation.

## Discussion

The ability of cells to sense the mechanical properties of their microenvironment and respond is a key determinant of cell behavior. The stiffness of the ECM itself is an important mechanical property. Although the importance of the biophysical microenvironment is well-documented, the molecular signaling events that relay that information remain incompletely understood. Here, we demonstrate that RasGRF1/Ras signaling regulates the survival of lung fibroblasts in response to substrate stiffness by regulating the activity of FOXO3a and Bim.

Ras activity has long been established as a major regulator of cell survival. Ras transformation results in the ability of the cells to undergo anchorage-independent growth, a key event in oncogenic transformation (6). Our study demonstrates that the enhanced survival seen in lung fibroblasts on stiff substrates is due to the activation of the small GTPase Ras. Ras activity is higher in cells plated on stiff substrates. If we genetically manipulate Ras activity, we found that constitutively active Ras will enable cells to survive on a soft substrate, while a dominant negative Ras will inhibit survival on a stiff substrate. These data are in agreement with work demonstrating that H-Ras expression allows fibroblasts to survive on soft substrates (13). More recent work demonstrates that expression of oncogenic K-Ras allows cells to sense the stiffness of the ECM and alter transcriptional events, suggesting that an increase in the ECM rigidity of the microenvironment is sufficient to initiate oncogenic cell reprogramming (14). Collectively, these studies identify Ras as a regulator of cell survival in response to mechanical stimuli from the microenvironment.

We also identify RasGRF1 as the GEF responsible for Ras activity on stiff substrates. RasGRF1 was first identified as a 140-kDa protein in the brain and has important functions in learning and memory (23, 24). Therefore, a number of studies have focused on the role of RasGrf1 in neuron function and neurite outgrowth (23). However, additional alternative splice variants of RasGRF1 have been identified in various tissues and cell lines (25, 26, 27, 28), including a splice variant of ∼43 to 50 kDa in the lung (27). In agreement with this observation, we find that MRC5 lung cells express a ∼43 to 50 kDa form of RasGRF1. Alternative forms of RasGRF1 are known to both activate Ras and initiate downstream signal transduction (28). In agreement with this observation, we find that lung RasGRF1 binds Ras, as evidenced by its binding to the RasG15A construct. We further find that lung RasGRF1 activity is associated with a concomitant increase in Ras activity. Moreover, siRNA against RasGRF1 abbrogates expression of the RasGRF1 lung variant and inhibits the activation of Ras. We also observe that lung RasGRF1 activity regulates signal transduction downstream of Ras.

Interestingly, several studies also point to a role for RasGRF1 in regulating Ras activity during mechanosensitive processes. RasGRF1 activity enhances cardiac fibrosis in the aging mouse heart (29) as well as increasing ECM deposition in the myocardium during diabetic cardiomyopathy (30). The idea that RasGRF1 activity contributes to the fibrotic phenotype is consistent with our results demonstrating increased RasGRF1 activity on stiff ECM. We also found that stiffness-induced RASGRF1 activity resulted in the activation of the downstream ERK and AKT pathways, which were critical in regulating survival on stiff ECM. This is in agreement with studies demonstrating activation of ERK and AKT in response to elevated RasGRF1 activity in multiple cell types (30, 31).

Mechanical signals are known to control transcription (1, 32, 33) but the precise signaling cascades that link the cellular microenvironment to transcriptional events are still being determined. One of the best established prosurvival pathways downstream of AKT involves the phosphorylation of the transcription factor FOXO3a. Regulation of FOXO3a activity is achieved by changes in subcellular localization, modulated by phosphorylation events, with transcriptional activity occurring along with nuclear localization (18). Activated AKT directly phosphorylates FOXO3a resulting in nuclear export and sequestration in the cytoplasm (34). In accordance with this model, we found that cells plated on rigid ECM showed enhanced phosphorylation of FOXO3a that was dependent on both RasGRF1 and AKT activity. This suggests that alterations in ECM stiffness are able to impact transcriptional events regulated by FOXO3a. Other transcriptional factors have also been linked to ECM stiffness. Phosphorylation and localization of YAP/TAZ are modulated by ECM stiffness through the Hippo pathway (35, 36, 37, 38). Also, in lymphatic endothelial cells, GATA2 transcriptional activity is altered by changes in ECM stiffness (39).

Interestingly, both FOXO3a and Hippo/YAP signaling have been linked to pulmonary fibrosis. FOXO3a is a critical regulator of profibrotic signaling in both a mouse model of idiopathic pulmonary fibrosis and in patient idiopathic pulmonary fibrosis samples (40). Hippo effectors YAP/TAZ are also activated in idiopathic pulmonary fibrosis (41, 42) and matrix stiffness regulates their localization (41). This raises the interesting possibility of crosstalk between the Hippo and FOXO3a pathways in diseases with altered matrix stiffness. Consistent with this idea, crosstalk between Hippo/YAP and another member of the FOXO family, FOXO1, has been reported (43).

FOXO3a transcriptional activity promotes the expression of proapoptotic Bim, while reducing expression of antiapoptotic Bcl-2 family genes (44, 45) making it a clear regulator of cellular survival. Our data demonstrate that the enhanced Ras activity observed on stiff substrates leads to the AKT-dependent phosphorylation of FOXO3a, thereby reducing its transcriptional activity, leading to a decrease in the expression of Bim. Interestingly, Bim is also a critical component of the cellular anoikis signaling cascade (46). The loss of cell adhesion during anoikis causes an accumulation of Bim, which normally undergoes proteasomal degradation upon integrin engagement (46). In adherent cells, ERK phosphorylates Bim at several sites and this phosphorylation targets Bim for ubiquitination and proteasomal degradation (47, 48). Similarly, we found that enhanced ECM stiffness results in a decrease in Bim expression. Likewise, we found that the remaining Bim protein levels showed increased ERK-dependent phosphorylation at S69, marking Bim for proteasomal degradation. These data suggest that perhaps some of the pathways initiated during anoikis may be due to the loss of mechanical signals from the microenvironment a cell experiences upon detachment.

Our study defines a molecular signaling pathway that translates ECM stiffness into cell survival through the activation of Ras *via* the GEF RasGRF1, to the activation of the PI3K/AKT and MEK/ERK pathways, resulting the phosphorylation of FOXO3a and the degradation of Bim. Understanding how these physical forces are transduced into cellular signaling cascades is a crucial first step in defining mechanical biomarkers and targets for cancer, cardiovascular disease, and fibrosis.

## Experimental procedures

### Cell culture

MRC5 cells were obtained from the American Type Culture Collection and grown in high glucose Dulbecco's modified Eagle's medium supplemented with 10% fetal bovine serum and antibiotic-antimycotic solution (Thermo Fisher Scientific). For substrate rigidity experiments, cells were cultured on hydrogels of 1 kPa or 50 kPa (Matrigen) coated with collagen (20 μg/ml) or fibronectin (20 μg/ml) for 24 h prior to harvesting.

### Statistical analysis

Statistical differences between two groups of data were analyzed with a two-tailed unpaired Student’s *t* test. All graphical data are represented as a mean with data bars representing SEM. A *p* value of <0.05 was considered statistically significant. Significance is denoted by ∗*p* < 0.05, ∗∗*p* < 0.01, ∗∗∗*p* < 0.001, and ∗∗∗∗*p* < 0.0001.

### Antibodies and inhibitors

Antibodies against the following proteins were used: AKT (2920, Cell Signaling Technology), phospho-AKT (9271, Cell Signaling Technology), phospho-ERK (9106, Cell Signaling Technology), Bim (2933, Cell Signaling Technology), phospho-Bim S69 (4584, Cell Signaling Technology), FOXO3a (2497, Cell Signaling Technology), phospho-FOXO3a (8174, Cell Signaling Technology), Sos (12409, Cell Signaling Technology), GAPDH (5174, Cell Signaling Technology) and Ras (3965, Cell Signaling Technology), ERK2 (sc154, Santa Cruz), RasGRF1 (ab190081, Abcam), RasGRF2 (SAB2700396, Sigma), and RapGEF2 (PA5-49456, Invitrogen). U0126 was purchased from Cell Signaling Technology, LY294002 was purchased from Tocris, and MG-132 was purchased from Calbiochem.

### Crystal violet staining

Cells were plated on soft or stiff hydrogels for 24 h and then fixed with 3.7% paraformaldehyde for 15 min. Cells were washed with PBS and incubated with 0.05% crystal violet solution for 20 min. Cells were washed with dH2O to remove excess dye. Cells were then visualized by light microscopy.

### Live/dead assay

Cell viability was measured using a fluorescent live/dead cell assay (Invitrogen), where calcein AM is used to detect living cells and ethidium homodimer (EtD-1) is used to detect dead/dying cells. After staining cells were observed microscopically. Cell death was quantified by determining the percent of EtD-1–positive cells to total cell number (EtD-1 and calcein-AM–positive cells). For the adenoviral Ras mutant experiments, GFP fluorescence was used to determine the total number of cells. A minimum of 300 cells across 10 fields were counted for analysis.

### siRNA transfections

MRC5 cells were transfected with siRNAs using Oligofectamine (Invitrogen) according to manufacturer’s protocol. Cells were cultured for 72 h posttransfection before final processing. The following siRNAs (Dharmacon) were utilized at 25 nM: nontargeting control: UAGCGACUAAACACAUCAAUU, RasGRF1: CCAACUAAAUCUCCAACAA and GAGAGAGGAGUCAGAUAUU, Bim: GCGGAGAAAUCAAGUUUAA and GGAAGUUUGUUGUGAAUGU, FOXO3a GCACAGAGUUGGAUGAAGU, and GUACUCAACUAGUGCAAAC.

### Adenoviral infection of Ras mutants

Adenoviral GFP, Ras N17, and Ras V12 were the kind gift of Dr Kevin Pumiglia (Albany Medical College) and were generated as described previously (49). Infection efficiency was monitored under a microscope by visualizing the coexpressed GFP marker. For experiments, MRC5 cells were infected with Ras adenoviruses for 24 h. The cells were then replated onto 1 kPA and 50 kPa Matrigen dishes for an additional 24 h before processing for the Live/Dead Assay.

### Ras activity assay

Cells were lysed for analysis of Ras (10 mM MgCl_2_, 500 mM NaCl, 50 mM Tris, pH 7.6, 1% Triton X-100, 0.1% SDS, 0.5% deoxycholate, 1 mM PMSF, and 10 μg/ml aprotinin and leupeptin) and cleared at 14,000*g* for 5 min. Lysates were incubated with glutathione-Sepharose–bound glutathione-*S*-transferase–Raf1-binding domain for 30 min at 4 °C with gentle rocking. Beads were then washed three times in 50 mM Tris, pH 7.6, 10 mM MgCl_2_, 150 mM NaCl, 1% Triton X-100, 1 mM PMSF, and 10 μg/ml aprotinin and leupeptin. Released proteins and reserved input control were subjected to Western blot analysis as described later.

### Nucleotide-free Ras pull-down assays

Affinity precipitation of exchange factors with the nucleotide-free Ras mutant (G15A) was performed as previously described Enzymology paper). Briefly, cells were lysed in 20 mM Hepes (pH 7.6), 150 mM NaCl, 1% Triton X-100, 5 mM MgCl2, 200 μM orthovanadate plus protease inhibitors. Equalized and clarified lysates were incubated with 20 μg of purified Ras(G15A) bound to glutathione-sepharose beads for 60 min at 4  °C. Samples were then washed, boiled in lysis buffer, and processed for SDS–PAGE.

### Western blotting

Samples were resolved on polyacrylamide gels in the presence of SDS. Resolved gels were transferred onto nitrocellulose membranes, blocked with 5% bovine serum albumin in Tris-buffered saline (25 mM Tris, pH 7.6, 150 mM NaCl) plus 0.1% Tween-20 (TBST) and incubated with primary antibody overnight at 4 °C with gentle rocking. Blots were washed extensively in TBST before being incubated with species-appropriate horseradish peroxidase-conjugated secondary antibody (Jackson Laboratories) for 1 h at room temperature. Blots were again washed in TBST, and fluorescence was detected using enhanced chemiluminescent reagent (Thermo Fisher Scientific) and X-ray film.

## Data availability

The original data are available upon request.

## Supporting information

This article contains supporting information.

## Conflict of interest

The authors declare that they have no conflicts of interest with the contents of this article.
