# Supplementary material for: ECM stiffness regulates lung fibroblast survival through RasGRF1-dependent signaling
Source: J Biol Chem. 2025 Jan 8;301(2):108161. doi: 10.1016/j.jbc.2025.108161 (PMC11835592; doi:10.1016/j.jbc.2025.108161)
Supplement: Supporting information [file mmc1.pdf]

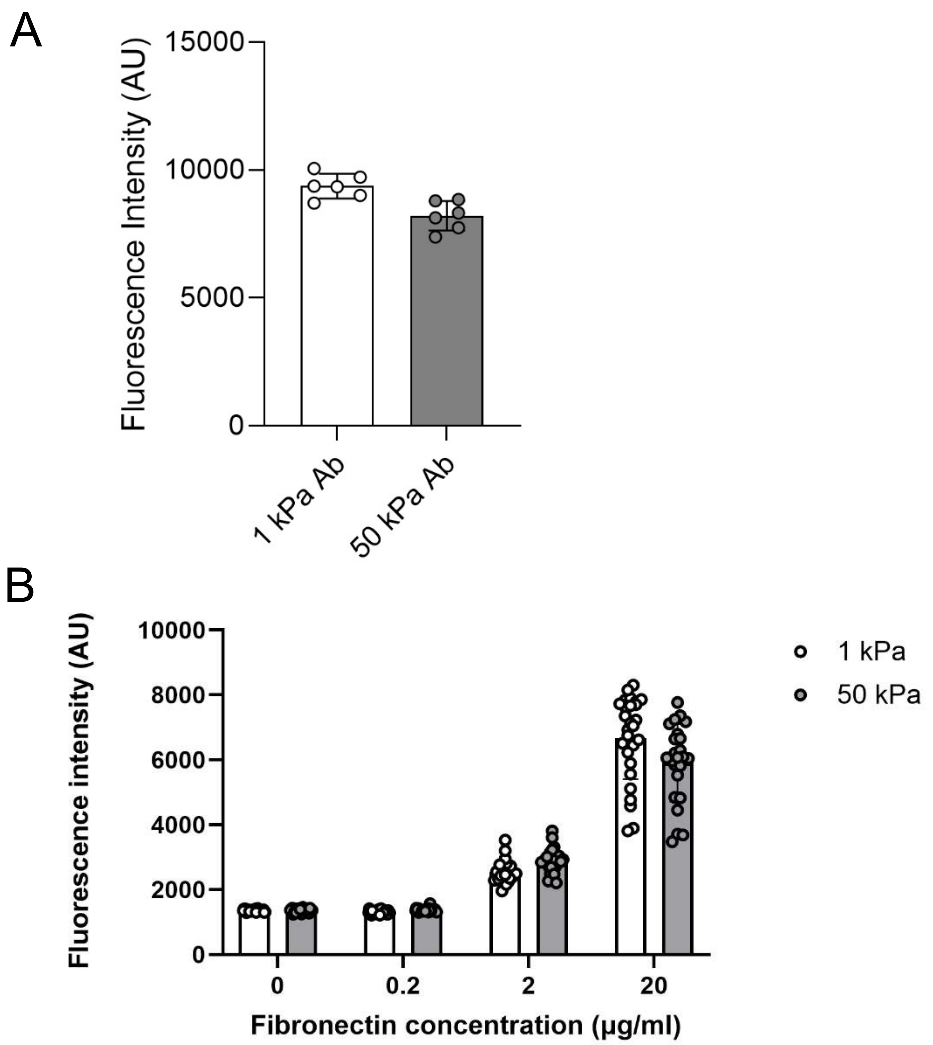

Fig S1. Relative ECM molecule binding to soft and stiff hydrogels. A) 1kPA and 50kPA hydrogels coated with 20ug/ml collagen (as supplied by manufacturer) were incubated with an Alexa Fluor 488 conjugated collagen antibody for 1 hour. Relative fluorescence intensity was determined. B) 1kPA and 50 kPA hydrogels were coated with increasing concentrations of Alexa Fluor 488-labelled FN for 1 hour. Relative fluorescence intensity was determined.
